# Very low protein diets supplemented with keto-analogues in ESRD predialysis patients and its effect on vascular stiffness and AVF Maturation

**DOI:** 10.1186/s12882-016-0347-y

**Published:** 2016-09-20

**Authors:** Cristiana David, Ileana Peride, Andrei Niculae, Alexandra Maria Constantin, Ionel Alexandru Checherita

**Affiliations:** 1 Clinical Department No. 3, “Carol Davila” University of Medicine and Pharmacy Bucharest, 37th Dionisie Lupu Street, 020021 Sector 2, Bucharest, Romania; 2 Department of Nephrology and Dialysis, “St. John” Emergency Clinical Hospital Bucharest, Bucharest, Romania; 3 Bucharest University of Economic Studies, Bucharest, Romania

**Keywords:** End-stage renal disease, Arterial stiffness, Arteriovenous fistula maturation, Ketoacid analogues of essential amino acids

## Abstract

**Background:**

Native arteriovenous fistula (AVF) is the most appropriate type of vascular access for chronic dialysis. Its patency rates depend on vascular wall characteristics. Ketoacid analogues of essential amino acids (KA/EAA) are prescribed in end-stage renal disease (ESRD) pre-dialysis patients to lower toxic metabolic products generation and improve nutritional status. We hypothesized that very-low protein diet (VLPD) supplemented with KA/EAA may influence arterial wall stiffness and affect AVF maturation rates and duration in pre-dialysis ESRD patients.

**Methods:**

In a prospective, cohort, 3 years study we enrolled 67 consecutive non-diabetic early referral ESRD patients that underwent AVF creation in our hospital. Patients were divided in two groups based on their regimen 12 months prior to surgery: a VLPD supplemented with KA/EAA *study group* versus a low protein diet non-KA/EAA-supplemented *control group*. For each patient we performed serum analysis for the parameters of bone mineral disease, inflammation and nutritional status, one pulse wave velocity (PWV) measurement and one Doppler ultrasound (US) determination prior the surgery, followed by consequent Doppler US assessments at 4, 6, 8 and 12 weeks after it. Rates and duration of mature AVF achievement were noted. We used logistic regression to analyze the association between AVF maturation and KA/EAA administration, by comparing rates and durations between groups, unadjusted and adjusted for systolic blood pressure, C-reactive protein, PWV, phosphorus values. All parameters in the logistic model were transformed in binary variables. A *p*-value < α = 0.05 was considered significant; data were processed using SPSS 16 software and Excel.

**Results:**

In the study group (*n* = 28, aged 57 ± 12.35, 13 females) we registered better serum phosphate (*p* = 0.022) and C-reactive protein control (*p* = 0.021), lower PWV (*p* = 0.007) and a higher percent of AVF creation success (33.3 % versus 17.8 %, *p* < 0.05). AVF maturation duration was lower in study group (5.91 versus 7.15 weeks, *p* < 0.001).

**Conclusions:**

VLPD supplemented with KA/EAA appear to improve the native AVF primary outcome, decreasing the initial vascular stiffness, possible by preserving vascular wall quality in CKD patients through a better serum phosphate levels control and the limitation of inflammatory response.

## Background

Autogenous arteriovenous fistulas (AVFs) are the preferred vascular access for chronic hemodialysis because of better outcomes, longer patency, lower infectious and mechanical complications rates, reduced costs and better survival for dialysis patients compared with prosthetic arteriovenous grafts (AVGs) or central venous catheters (CVC) [1–3].

Although agreed that the quality of the vascular access is crucial for achieving an adequate dialysis and a plurality of programs to improve its patency rates are developed, native AVFs are not easy to obtain. Their primary failure rates, due to maturation failure and stenotic complications, are reported to be between 10 and 60 % [1, 3–5]. Successful maturation depends on the initial diameter of the vessels and the abilities of the artery and the vein to develop flow-mediated dilatation and remodeling [5–7]. These characteristics appear to be determined by vascular health factors: wall elasticity and the endothelial response to increased flow and wall shear stress expressed by the release of vasodilators such as nitric oxide [8–10]. Some studies did not find any relation between the vessels dilatation capacity and AVF maturation rates, so the subject is still under debate [11–13].

The beneficial effects of ketoacid analogues of essential amino acids supplements (KA/EAA) in the nutrition of end-stage renal disease (ESRD) pre-dialysis patients are highlighted by many randomized controlled trials [14–21]. They reduce the rate of progression of chronic kidney disease (CKD) and decrease inflammation, lowering the phosphate absorption and can maintain a good nutrition status even when associated with very low-protein diets (VLPD) [14–22]. In our knowledge, there are no studies assessing the relation between KA/EAA supplements prescription and AVF success rates or maturation time. One trial made by Duenhas et al. describes a reduction of emergency access complications due to the delay in the dialysis initiation provided by KA/EAA supplements [22].

*We hypothesized* that ESRD patients complying with a VLPD supplemented with KA/EAA in pre-dialysis should have an improved arterial wall elasticity and better AVF maturation rates and durations, as this regime may have effects on relieving some of the predisposing factors of the vascular stiffness (phosphorus and parathormon (PTH) levels, inflammatory promoters). This study’s *primary objective* was to examine if there is an effect of VLPD supplemented with KA/EAA on success rates and maturation duration of native AVF in ESRD patients referred to our surgery department. *Secondary objectives* included pre-surgery pulse wave velocity (PVW) assessment as a marker of arterial stiffness, and the evaluation of biochemical parameters of interest for vascular wall quality in pre-dialysis KA/EAA supplemented patients undergoing AVF formation.

## Methods

During a three years period (January 2012 to January 2015) all ESRD patients (glomerular filtration rate (GFR) < 15 mL/min/1.73 m^2^), 18 years or older, that underwent AVF creation in the Department of Vascular Surgery of our hospital were recorded, at the moment when the appointment for surgery was made. Only the patients that proved monthly monitoring (based on personal medical reports and monthly prescriptions recorded in our hospital electronic data base) for a period of minimum 12 months prior the surgery (named early referral patients) were recruited for the screening visit. Diseases and conditions that could interfere with the intrinsic vascular wall properties were excluded: diabetes mellitus, serum albumin < 3.5 mg/dL, C-reactive protein (CRP) > 6 mg/L and history of cancer, chemotherapy and/or immunomodulatory therapy, peripheral vascular disease. Sixty seven patients were enrolled, based on inclusion criteria and after signing an informed consent for willingness to participate. The study was approved by the Local Ethical Committee of our Emergency Clinical Hospital (*No. 14570/10.01.2012*).

According to the medical history records, the patients were divided in two groups based on their pre-dialysis diets prescribed by their primary nephrologists and agreed by them: the KA/EAA positive group (VLPD and KA/EAA supplements prescriptions for minimum 12 months) – the *study group* (*n* = 28); the KA/EAA negative group (low protein diet and no KA/EAA supplements) – *control group* (*n* = 39). The KA/EAA available in our country is Ketosteril (*Fresenius Kabi*) in 630 mg per tablet. Paper forms were made for every patient with an affidavit indicating the regime they followed and the compliance to the treatment (with five options ticked choice: 0, 25, 50, 75, 100 % compliance); medical reports of the prescriptions for KA/EAA were collected from hospital’s electronic archive (in Romania, KA/EAA treatment in ESRD patients is reimbursed).

Our department’s guidelines recommend that CKD patients with GFR < 15 mL/min/1.73 m^2^ are to be prescribed a protein ingestion of 0.6–0.8 g/kg body weight/day without KA/EAA supplementation or 0.4–0.6 g protein/kg body weigh/day with KA/EAA supplementation (Ketosteril, 1 tablet/5 kg BW/day). The prescriptions were made by the nephrologists who monitored the patients in the pre-dialysis period, blinded to the study, intending to maintain the nutritional biochemical parameters recommended by KDOQI (*Kidney Disease Outcomes Quality Initiative*) for ESRD patients [14]. According to the same KDOQI guidelines, patients from both groups received phosphate binders, calcium and vitamin D supplements when necessary.

The medical history was recorded and a set of biochemical tests was taken at *the first visit* (one week prior to the surgery), along with the PWV measurement and a Doppler ultrasound (Doppler US) evaluation. At one week after the first visit AVF were created, by the same surgeon. Pre-surgery US Doppler evaluations for AVF placement are a routine in our center for cases with unsatisfactory data on clinical examination. The intervention was considered suitable at an artery diameter > 2 mm, a vein diameter > 2.5 mm, and it respected the standard surgical procedure. In patients with poor quality distal vessels we approach proximal sites from the beginning. 1 % lidocaine is used for local anesthesia and we do not prescribe perioperative antibiotics. Our surgeon mostly uses the artery-side to vein-end technique, mobilizing the vein to adapt the artery. We do not ligate collateral veins in the creation session. When needed, the two-steps superficialization is performed: first the creation, than the transposition of the AVF in another separate session.

The management policy in our center for all the new created AVF is the clinical examinations at 1, 4 and, if needed, 6 weeks after the surgery. Doppler US is prescribed only in clinical inconclusive cases. As we are an initiation center for renal replacement therapy and we provide patients for several centers in and around Bucharest, further examinations usually are at the prescription of the center’s nephrologists.

For the patients enrolled in the study, visits 2, 3, 4 and 5 consisted in clinical and Doppler US evaluations at 4, 6, 8 and 12 weeks after the surgery. AVF maturation was defined with Doppler US measurements of diameter > 0.6 cm and access flow > 600 mL/min [3]; failure creation of the native AVF was considered the failed surgery and failed maturation was defined when the above mentioned criteria for success maturation were not fulfilled. When a patient fulfilled these criteria simultaneously, it was recorded as a mature AVF at that interval of time (4, 6, 8 or 12 weeks).

Biochemical determinations included albumin, calcium, phosphorus, creatinine, C-reactive protein and cholesterol levels, determinations made at hospital laboratory using a *Mindray* analyzer; for iPTH (intact parathormon) one private lab performing ECLIA (*Electrochemiluminescence Immunoassay*) determination was used.

PWV measurements were done with the validated oscillometric device Mobil-O-graph PWA device (*Industrielle Entwicklung Medizintechnik, Germany*) with incorporated *IEM-Hypertension Management Software*, handled by the same research technician who was blinded to the clinical data. The device measures central blood pressure (BP) values and displays them along with the PWV values. All the guidelines for this examination were respected [23]. Briefly, patients were placed in a quiet room, in the seated position, and the cuff of the device was placed on the left arm – brachial artery; data including age, weight and height, along with the smoker/nonsmoker status were introduced. The device makes two recordings for BP values and then it measures the velocity of arterial pulse wave based on the oscillations detected on the upper-arm cuff during systole and displaying it together with the normal range for each patient using the integrated PWV algorithm.

Doppler ultrasound was performed with the same device (Aloha Cardiology P.C.) by one cardiologist, with the patient in the upright seated position; two determinations were made in a session for a single data recording. To avoid turbulence determined accuracy variations, the flow was measured in the distal part of the access, with the transducer (a 7.0 MHz probe) in the longitudinal position; the diameter was measured with the transducer perpendicular to the vessel [3]. Although many studies recommend AVFs’ flow measurement in the feeding artery, we performed the diameter and flow measurements in the vein, distal to the anastomosis (>2 cm), since the venous walls’ characteristics are significant for a good access and the post-procedural complications develop mostly at this level (stenosis, sclerosis or other narrowing causes) [24, 25].

*The primary outcome* was to evaluate the differences of the AVF success creation rates and maturation durations between the study and the control group. *The secondary outcome* was to compare the arterial stiffness – evaluated using PWV determination – and biochemical profiles between the two groups.

### Statistical analysis

Baseline pre-surgery biochemical characteristics were compared between the two groups using Fisher test for categorical parameters (to validate data). Normal distributed values were analyzed using the mean values and standard deviation. Parameters expressed by percentages were also compared using Chi-squared test. We used logistic regression to analyze the association between AVF maturation and KA/EAA administration, by comparing the average AVF maturation duration between the two groups, unadjusted and then adjusted for systolic BP, C-reactive protein, PWV, and phosphorus values. All the considered parameters in the logistic model were transformed in binary variables. A *p* value < α = 0.05 was considered statistically significant, and data processing was performed using SPSS 16 software and Excel.

## Results

**Fig. 1:**
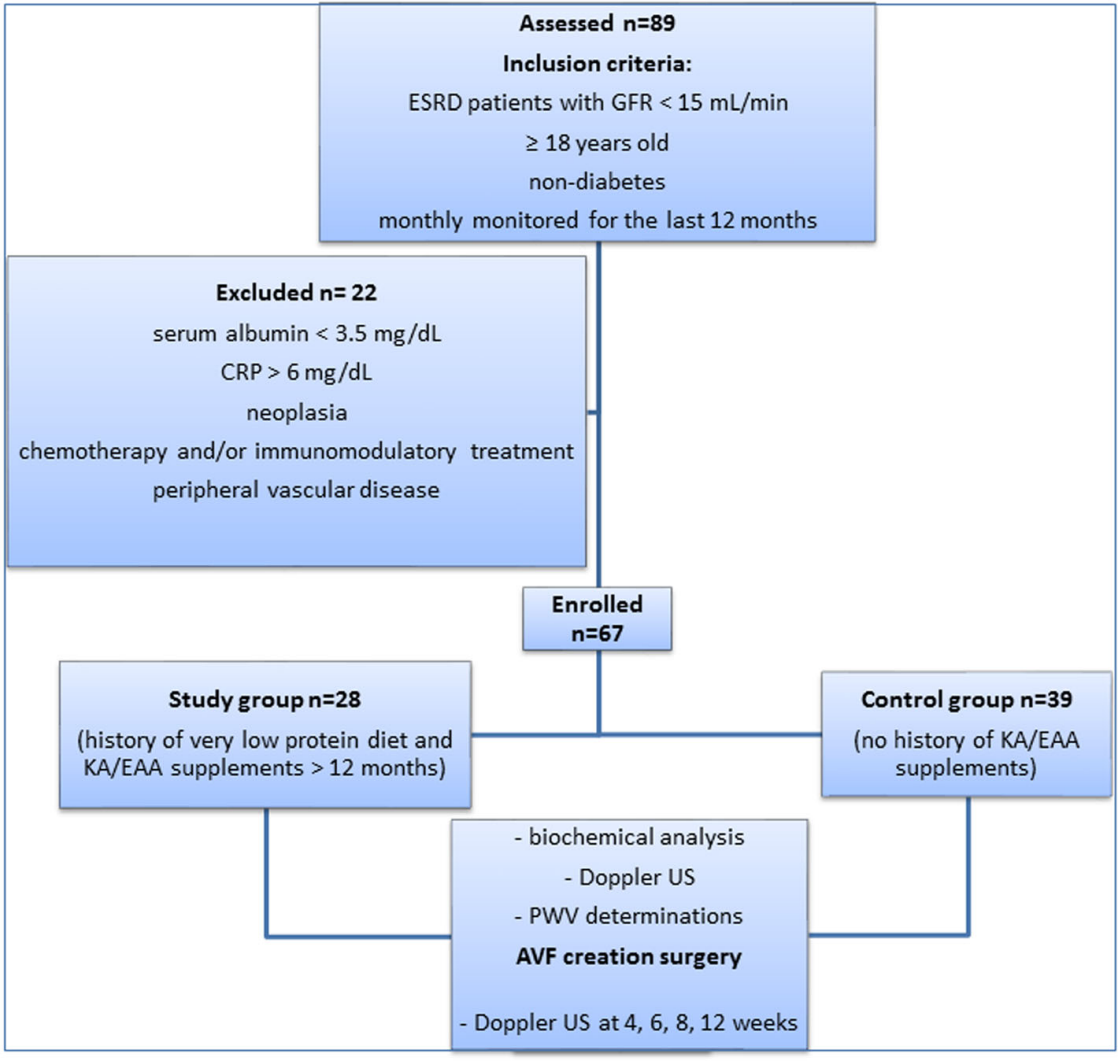
Enrollment chart and study design scheme

Sixty seven subjects were monitored until the end of this prospective observational study: 28 patients who received KA/EAA supplements for 12 months before the study enrollment (*study group*) and 39 patients without KA/EAA supplements in the last 12 months prior to study enrollment (*control group*). Three patients were lost to follow-up during the 3 months period and were excluded from the results. The diagram of patient’s enrollment scheme is shown in Fig. 1.

### Demographical data and other characteristics

Demographical data in the study and control groups are shown in Table 1, observing a mean age of 57 ± 12.35 years in the study group and 54 ± 11.7 in the control group, respectively (*p* < 0.001; SD = 2.471); additionally, 46.4 % in the study group were female, 48.7 % in the control group, respectively (*p* < 0.001; SD = 2.298). In this table we also showed other characteristics which can influence the arterial wall stiffness evolution and the PWV values – the number of smokers and the mean BP values (recorded during the PWV determination) in each group of patients. There was no significant difference regarding the percent of smokers in studied groups; BP values showed a significant difference between groups – *p* = 0.0141; SD =12.122.

**Table 1:** Demographical and biochemical characteristics compared in the groups

Characteristics	Study group (28 patients)	Control group (39 patients)
Demographics		
Age	57 ± 12.35	54 ± 11.7
Female	13 (46.4 %)	19 (48.7 %)
Other characteristics		
BMI	28.3 ± 6.13	28.1 ± 6.08
Smokers	8 (28.5 %)	10 (25.6 %)
Systolic BP (mmHg)	148 ± 7.79	154 ± 6.58
Vitamin D/VDRA (*n*)	6 (21.4 %)	11 (28.2 %)
Phosphorus binders (*n*)	0	5 (12.8 %)
Antihypertensive	21 (75 %)	32 (82 %)

*BMI* body mass index, *BP* blood pressure, *VDRA* vitamin D receptor activation

### AVFs outcomes

The primary outcome parameters – surgery results and characteristics of AVF maturation – are presented and compared between the two groups in Table 2.

**Table 2:** Vascular and dialysis initiation characteristics compared in the groups

Characteristics	Study group (28 patients)	Control group (39 patients)	*χ* ^2^ value	SD-value	*p*-value
Failed AVF creation cases	2 (7.1 %)	5 (12.8 %)	3.11	2.01	0.021
Failed AVF maturation cases	3 (10.7 %)	8 (20.5 %)	2.82	3.19	0.017
AVF diameter (cm) at 4 weeks	0.60 ± 0.09	0.51 ± 0.08	73.396	0.226	<0.001
AVF flow (mL/min) at 4 weeks	698.07 ± 136.13	579.12 ± 105.84	317.2	337.034	0.017
Upper arm AVF (*n*)	6 (21.4 %)	9 (23.1 %)			
AVF diameter (cm) at 3 months	0.70 ± 0.07	0.67 ± 0.08	84.35	0.191	<0.001
AVF flow (mL/min) at 3 months	756.92 ± 136.62	722.65 ± 122.25	364	338.25	0.007
Maturation time (weeks)	5.91 ± 0.92	7.15 ± 1.19	34.45	2.775	<0.001
Dialysis initiation (%)	11 (39.3 %)	19 (48.7 %)	6.31	5.61	0.5
CVC-HD initiation (%)	2 (7.1 %)	5 (12.8 %)	4.5	2.121	0.5

*AVF* arteriovenous fistula, *CVC*-*HD initiation* hemodialysis initiation on central venous catheter, *χ* ^2^ value > *χ* ^2^ critical value, statistically significant, *p*-value < α-value = 0.05, statistically significant

A success rate of 83.33 % was registered in the cohort, with an average maturation time of 6.57 +/− 2.64 weeks. Twenty two lower arm AVFs were performed in the study group – 6 radiocephalic (wrist) and 16 brachiocephalic fistulas (middle-arm). In the control group, 30 lower arm AVFs were performed – 7 radiocephalic (wrist) and 23 brachiocephalic fistulas (middle-arm). In 15 cases we were forced to perform upper arm fistulas – 6 in the study group (21.4 %) and 9 in the control group (23.07 %). In 6 cases we managed to save or improve the newly created AVF (thrombosis, stenosis or collateral veins) by surgical procedures during the study (two cases in the study group and four in the control group).

Eighteen patients failed to achieve a suitable vascular access for hemodialysis in the 3 months interval: 5 from the study group and 13 from the control group (*p* < 0.05). There were many significant differences between groups, regarding both surgery success and maturation rates and duration. The study group had only a 7.1 % of procedure failure compared with a 12.8 % unsuccessful surgery in the control group (*p* = 0.021). The percent of AVF’s which fulfilled maturation criteria in the control group was lower and the necessary period of time significantly longer than in the study group (*p* < 0.001).

Seven central venous catheters were placed for emergency initiation of renal replacement treatment in this period of time, of which only 2 were registered in the study group and the other 5 in the control group. In 4 patients (3 in the control group and 1 in the study group) there were other attempts for AVF creation, in proximal positions, but the patients were no longer recorded in our study.

The mean interval for starting dialysis in patients requiring renal replacement therapy during the study (30 patients) was: 50.454 ± 24.18 days in the study group (11 patients), respectively 49.263 ± 23.00 days in the control group (19 patients).

### Secondary outcomes – PWV values and biochemical determinations

The biochemical parameters found to be different between groups are C-reactive protein (*p* = 0.021) and serum phosphorus levels (*p* = 0.022), with similar statistical significance. All other average serum values were similar in study group and control group, including cholesterol (*p* = 0.311), iPTH (*p* = 0.269), creatinine (*p* = 0.2324), calcium (*p* = 1.703), albumin (*p* = 0.887).

The results for mean PWV values and serum biochemical determinations for each group are detailed in Table 3.

**Table 3:** Significant statistical differences between the studied groups (KA/EAA group and control group) regarding the analyzed parameters

Characteristics	Study group (28 patients)	Control group (39 patients)	*χ* ^2^ value	SD-value	*p*-value
Creatinine (mg/dL)	6.9 ± 0.16	7.0 ± 0.15	8.27	0.416	0.2324
Calcium (mg/dL)	9.1 ± 0.15	8.8 ± 0.11	3.867302	0.389	1.703
Phosphorus (mg/dL)	4.0 ± 0.19	5.2 ± 0.33	253.2	*0.608*	0.022
iPTH (pg/mL)	118 ± 25.56	129 ± 27.95	0.625	56.061	0.269
C-reactive protein (mg/L)	1.2 ± 0.38	2.3 ± 0.48	222.8	0.988	0.021
Albumin (g/dL)	4.2 ± 0.11	3.9 ± 0.10	13.93	0.278	0.887
Cholesterol (mg/dL)	206 ± 44.63	214 ± 46.17	0.48	44.35	0.311
PWV [90 % CI], (m/s)	9.39 ± 0.83	10.61 ± 1.07	513.5	2.741	0.007

*iPTH* intact parathormon, *PWV* pulse wave velocity, *χ* ^2^ value > *χ* ^2^ critical value, statistically significant, *p*-value < α-value = 0.05, statistically significant

The average calculated compliance on diet and treatment was 80.76 ± 23.77 in study group and 79.41 ± 23.41 in control group, with no statistic differences noted (*p* = 0.158).

### Elaborate statistic correlations

**Fig. 2:**
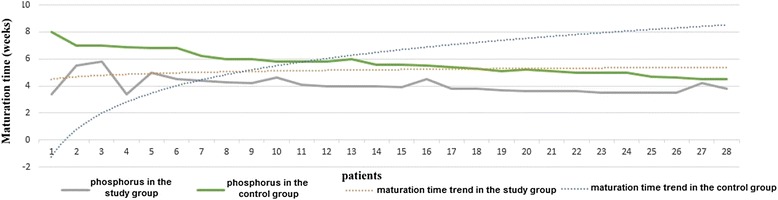
The influence of phosphorus values on AVF maturation time trend in both groups

**Fig. 3:**
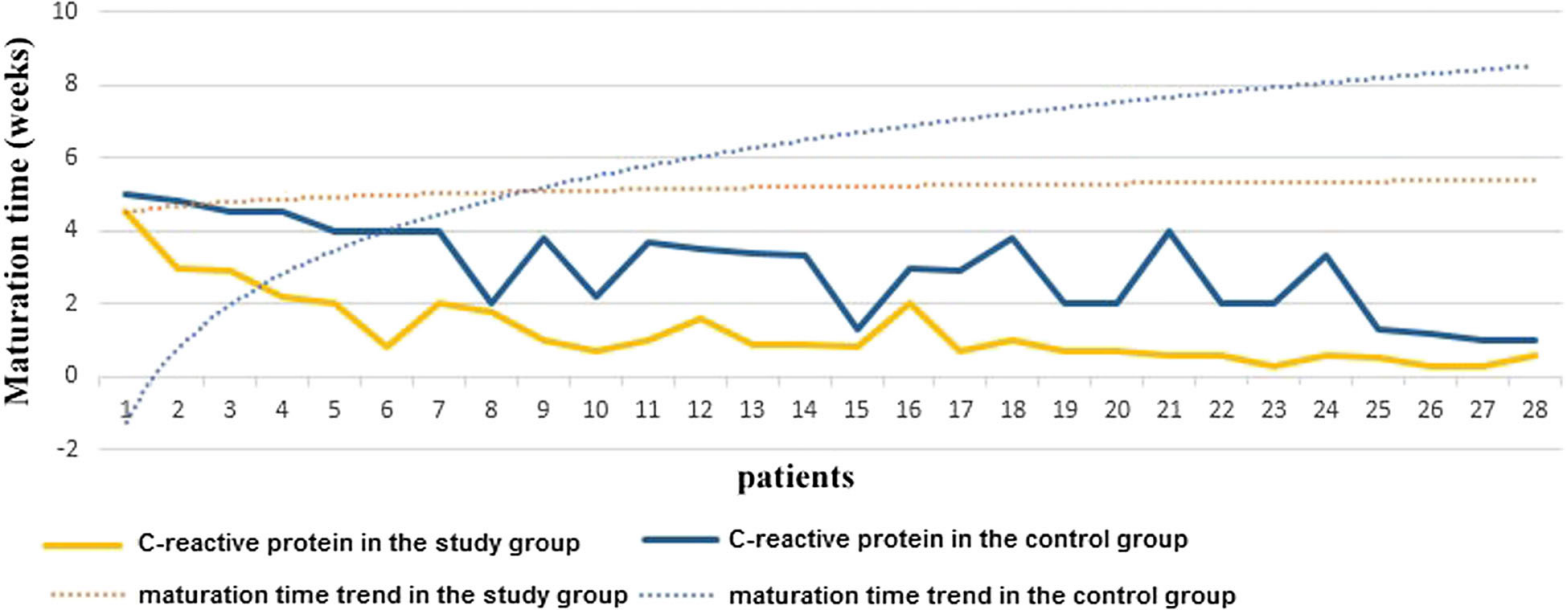
The influence of CRP values on AVF maturation time trend in both groups

C-reactive protein and phosphorus are the only biochemical parameters that have directly influenced maturation time, as it follows: in KA/EAA group, due to lower levels of phosphorus and CRP, the maturation time was shorter than in the control group (Figs. 2 and 3):

$$ MT= 3.891\times CRP+ 1.44 \times P\hbox{--} in\ KA/EAA\  group $$

$$ MT= 1.624\times CRP+ 6.123\times P\hbox{--} in\ non-KA/EAA\  group $$

*where MT* = *maturation time*; *CRP* = *C*-*reactive protein*; *P* = *phosphorus*.

In the control group, the logistic regression formula highlighted that increased phosphorus levels influenced AVF maturation time more profound than CRP values. In contrast, in KA/EAA group, CRP levels presented a more powerful contribution on the evolution of the AVF maturation period than phosphorus values.

When applying linear regression, regarding the influence of AVF diameter and flow on maturation time, at 4 weeks and 3 months, respectively, it could be demonstrated that, in the study group, the AVF diameter had a more significant impact on the maturation period than AVF flow, especially at 4 weeks. In contrast, in the control group, both AVF diameter and flow equally influenced the maturation time (Table 4).

**Table 4:** The influence of AVF diameter and flow at 4 weeks and 3 months on maturation time in both groups

Parameters	Study group (28 patients)		Control group (39 patients)	
	Coefficient	*p*-value	Coefficient	*p*-value
AVF flow at 4 weeks	0.582	0.415	0.876	0.013
AVF diameter at 4 weeks	−1.099	0.012	−1.330	0.001
AVF flow at 3 months	0.462	0.558	−0.063	0.822
AVF diameter at 3 months	−0.755	0.095	−0.371	0.232

*AVF* arteriovenous fistula

Furthermore, when comparing the influence of BP on PWV values (χ2 = 21.44, *p* < 0.001 in the study group, and 22.78, *p* < 0.001 in the control group), we noticed that BP had a higher influence on PWV levels in the control group than in the study group (0.143 versus *0.137* – *correlation coefficient*).

## Discussions

This study focuses on the impact of KA/EAA supplemented VLPD on the vascular wall quality and on the chances for a successful vascular access creation. At the moment of fistula surgery we analyzed the differences of several biochemical parameters considered at interest between a VLPD KA/EAA supplemented group of patients (*n* = 28) and a conventional low protein diet, non-KA/EAA, control group (*n* = 39). The repartition of the patients in the two groups was made based on their documented history (the registered prescriptions for keto-analogues, a reimbursed treatment for ESRD patients in our country). After the vascular access creation, we analyzed AVF characteristics and maturation process in all the surgery-successful patients (60 cases): *26 patients in the study group and 34 patients in the control group*.

There is no consensus on the criteria to define fistula maturation. We chose the KDOQI definition, according to which an adequate AVF has a flow > 600 mL/min and a diameter of the vein of > 0.6 cm [3, 26]. One third to over a half of fistula creation surgery procedures worldwide resides in failures – failure of creation, delayed maturation or early fistula failure [8, 13, 26, 27]. In our study, the percent of failure was 26.86 % in whole group, a reasonable percent considering that the study cohort was made of monthly monitored patients for at least one year before renal replacement therapy preparations. The AVF maturation rate was 83.33 % (50 patients) and the mean AVF maturation duration in the whole group was 6.57 ± 2.64 weeks, with pronounced differences between control group and the KA/EAA group (*p* < 0.001).

We also evaluated the situation of the vascular access at the 4 weeks measurement, when an AVF flow > 500 mL/min and a vein diameter after the anastomoses of > 0.4 cm can predict the future AVF adequacy [24, 28, 29]. At that point we noted that a difference already exists between the two groups, and it deepens at 6 weeks and at 12 weeks measurements. This is in contrast with the findings of Robbin et al. who determined no significant improvements in the development of AVF in the second and third months [24]. A gradual continuous increase in AVF blood flow until 12 weeks is sustained by other studies [8, 29, 30]. At the end of the study, a higher percent of failures in AVF creation was recorded in the non-KA/EAA control group (33.3 % versus 17.8 %). We propose some explanations for the differences registered between the studied groups.

For fistulas to mature, the vessels have to dilate: their diameters must increase by 40–60 % and their flows 10–20 times [5, 8, 12, 13, 30, 31]. In ESRD patients this process is hampered by an increased wall stiffness, due to medial thickening and calcifications plus intimal inflammation and hyperplasia [12, 13, 32–36].

The elasticity of the vessel wall is defined by its characteristics: the quality of its constituents, de degree of the stiffness and vascular calcifications [8, 37]. Arterial stiffness is recognized as an important factor of vascular health [38]. Its levels were widely analyzed in relation to the cardiovascular risk in CKD patients [39]. There are many indicators proposed to predict the dilatation capacity and, through it, the AVF success: PVW, peripheral arterial tonometry (PAT), flow mediated dilation (FMD). None of them proved their supremacy [40]. PWV is the most important indicative for global vascular stiffness but it was rarely explored in ESRD patients as a predictor of AVF success.

We determined PWV values in both groups of patients and we found them lower than other data from the literature, probably due to the enrollment policy – early referral patients monitored for minimum 12 months [41, 42]. Nevertheless, values were significantly different between the two groups (*p* = 0.007). It appears that VLPD and KA/EAA administration had a protective effect against vascular stiffness.

Analyzing the biochemical parameters list, the variable that is significantly different between groups and can be associated with arterial stiffness by attending vascular calcifications is the serum phosphorus level. Phosphorus plays a central role as a promoter in vascular inflammatory processes, affecting vascular wall elasticity and stimulating vascular calcifications [43–45]. VLPD and KA/EAA supplements ameliorate calcium-phosphate deregulations in ESRD by lowering serum phosphorus levels through several mechanisms. Initially, the smaller the protein intake, the lower the amount of phosphates provided for absorption. Secondly, the calcium salts of keto analogues in the KA/EAA composition acts like phosphate binders and hamper phosphorus absorption; in the same time they provide the necessary calcium supplementation for the hypocalcemia of CKD [46]. A third possible way of action, the protective effect of KA/EAA supplements against metabolic acidosis, is yet to be further studied, considering that acidosis is an important contributor to vascular wall pathology in ESRD [47, 48].

Incomplete compliance to diet and treatment was noted in both groups in similar proportions, but we considered that exceeding the limits permitted in VLPD resulted in relatively smaller amounts of ingested proteins (and phosphates) that in low/normal protein diet. In addition, the greater need of phosphate binders in the control group resulted in an increased susceptibility of treatment noncompliance in this group, as this medication is effective when taken with meals and it can change the taste of food.

The significantly lower phosphate levels observed in the KA/EAA group appears as an important feature; there is a recent opinion that *low protein diet* accompanied by a *high serum phosphate* level is likely to increase vascular calcifications through calcium-phosphate precipitates and inflammation promotion [49]. The phosphate binding property of KA/EAA supplements is an important part of its beneficial effects and necessary complement to VLPD [46].

CRP was found near the non-risk limit in the KA/EAA group (1.1 mg/L) and significantly increased in the control group, showing a predisposition to atherosclerotic vascular disease, endothelial alterations and vascular stiffness in non-treated patients [50–53]. This beneficial effect of KA/EAA supplements on inflammation markers (CRP, adiponectin etc.) was also demonstrated by Chen et al. [20].

Because of the retrospectively nature of nutritional assessment of our research we could not gather precise data for estimating protein-energy wasting (PEW) in our study groups. We can only rely on other studies that pointed out a benefic effect of the VLPD supplemented with KA/EAA in reducing the severe phenomenon of PEW in non-dialysis ESRD [50, 51, 54–58]. Oxidative stress, inflammation and insulin resistance contribute at PEW in CKD patients [57, 59–62]. By reducing the inflammation and maintaining a better protein balance, KA/EAA may decrease PEW and improve vascular wall quality [57–62].

It is proved that a VLPD supplemented with KA/EAA produces decreased levels of toxic metabolic substances and lowers the catabolic tendency of CKD, protecting against oxidative stress [59, 63]. A benefic effect on indoxyl sulfate serum levels was pointed out by other studies that emphasizes the KA/EAA supplements role in decreasing oxidative stress and endothelial dysfunction when prescribed in stage 3 and 4 of CKD [58, 64, 65].

There are a few limitations of this study. First is the exclusivist selection of the patients. In order to limit the influences of representative comorbidities upon the vessel wall and having an accurate picture of the VLPD and KA/EAA vascular action, we selected only non-diabetes patients and excluded severe malnutrition and inflammation. However, diabetic nephropathy is the most important primary disease for CKD and further surveys including diabetic patients are needed to establish the influence of pre-dialysis KA/EAA supplementation in this particular type of chronic renal patients.

Another debatable characteristic of our study is the wide range of BP values at which the PWV measurements were performed. Although it was emphasized that the impact of BP upon PWV values was influenced by very-low-protein diet and KA/EAA supplementation, we admit that when performing PWV measurements in patients with better controlled systolic BP values (below 160 mmHg), an improvement of the test accuracy can be achieved.

Last, but not least, it is a limitation about the mode we establish the compliance to treatment and diet for each patient, on the basis of retrospective data and patients’ statements; the KA/EAA supplementation was prescribed and monitored by the patients’ nephrologists, until the enrollment moment in the study. Urinary urea levels were not measured. A prospective study, starting with registered baseline data and controlled assignment in two groups – VLPD supplemented with KA/EAA group and control group – should be done, in order to certify the outcomes of chronic vascular access in treated and non-treated patients.

Recognizing its limitations, this is the first study demonstrating that VLPD supplemented with KA/EAA is effective in rising the percent of successful AVF creation and shortening the duration of fistula maturation, with positive influences on arterial stiffness.

A prospective randomized controlled study is our agenda, in which we intend to enroll a larger number of patients, including diabetics. We plan to obtain wall vessel specimens during the AVF creation surgery, analyze their morphological structure and compare the results between the two groups of patients – the low-protein diet and the supplemented very-low-protein diet groups.

## Conclusions

Achieving a reliable native vascular access is a major desiderate in ESRD patients and it must be prepared from early pre-dialysis stages. VLPD supplemented with KA/EAA appear to improve the native AVF primary outcome, decreasing the initial vascular stiffness, possible by preserving vascular wall quality in CKD patients through a better serum phosphate levels control and the limitation of inflammatory response.

Further studies with larger sample size and vascular endothelial exploring are required in order to deepen understanding mechanisms underlying these effects and certify the role of VLPD supplemented with KA/EAA in relieving vascular stiffness and ameliorating AVFs maturation rates and durations.

## Abbreviations

AVFs: Arteriovenous fistulas
AVGs: Arteriovenous grafts
BMI: Body mass index
BP: Blood pressure
CKD: Chronic kidney disease
CRP: C-reactive protein
CVC: Central venous catheters
CVC-HD initiation: Hemodialysis initiation on central venous catheter
Doppler US: Doppler ultrasound
ECLIA: Electrochemiluminescence immunoassay
ESRD: End-stage renal disease
FMD: Flow mediated dilation
GFR: Glomerular filtration rate
iPTH: Intact parathormon
KA/EAA: Ketoacid analogues of essential amino acids supplements
KDOQI: Kidney Disease Outcomes Quality Initiative
PAT: Peripheral arterial tonometry
PEW: Protein-energy wasting
PTH: Parathormon
PVW: Pulse wave velocity
VDRA: Vitamin D receptor activation
VLPD: Very low-protein diets
